# Temporal changes in corticosteroid dose during ibrutinib treatment in patients with cGVHD and pulmonary involvement

**DOI:** 10.1007/s12185-024-03882-1

**Published:** 2024-12-10

**Authors:** Masako Toyosaki, Shinichiro Machida, Daisuke Tomizawa, Masaya Okada, Masashi Sawa, Yasunori Ueda, Ai Omi, Yosuke Koroki, Takanori Teshima

**Affiliations:** 1 Department of Hematology and Oncology, Tokai University Hospital, Isehara, Japan; 2 Division of Leukemia and Lymphoma, Children’s Cancer Center, National Center for Child Health and Development, Tokyo, Japan; 3 Division of Hematology and Oncology, Kansai Medical University General Center, Osaka, Japan; 4 Department of Hematology and Oncology, Anjo Kosei Hospital, Anjo, Japan; 5 Department of Hematology/Oncology, Kurashiki Central Hospital, Kurashiki, Japan; 6 Medical Affairs Division, Janssen Pharmaceutical K.K, 3-5-2 Nishi-kanda, Chiyoda-ku, Tokyo, 101-0065 Japan; 7 Department of Hematology, Faculty of Medicine, Hokkaido University, Sapporo, Japan

**Keywords:** Chronic graft-versus-host disease, Corticosteroids, Ibrutinib, Japan, Lung

## Abstract

The GVH3001 study assessed the efficacy and safety of ibrutinib in Japanese patients with steroid-dependent or -refractory chronic graft-versus-host disease (cGVHD). However, the effects of ibrutinib on lung function and reduction in corticosteroid dose, which is a measurable factor associated with improved quality of life, could not be adequately assessed in patients who initially presented with lung involvement. This *post hoc* analysis aimed to evaluate temporal changes in daily corticosteroid dose, as well as effectiveness outcomes based on lung function and symptom burden (percent predicted forced expiratory volume in 1 s [%FEV_1_] and Lee cGVHD Symptom Scale lung subscale score, respectively) in the subgroup of patients with cGVHD who had lung involvement at baseline. Seven of the 19 patients in the GVH3001 study had lung involvement at baseline. The daily corticosteroid dose for cGVHD decreased in five of these patients, and %FEV_1_ remained relatively stable in two patients but increased to > 80% in one patient. Lee cGVHD Symptom Scale scores were relatively stable throughout the study in patients with lung involvement. Ibrutinib may allow corticosteroid dose reduction without worsening lung function or increasing symptom burden in previously treated patients with cGVHD and associated lung involvement.

**Supplementary Information:**

The online version contains supplementary material available at 10.1007/s12185-024-03882-1.

## Introduction

Chronic graft-versus-host disease (cGVHD) is a serious and potentially life-threatening complication of allogeneic haematopoietic stem cell transplantation (HSCT) [1], which can have a significant impact on a patient’s quality of life (QoL) [2]. Between 30 and 70% of patients globally who undergo HSCT develop cGVHD [1–4]. In Japan, the 2-year cumulative incidence of cGVHD was estimated at 35.4% in 2012–2014 [5]. The management of cGVHD in Japan corresponds with international practice, where both corticosteroids and calcineurin inhibitors are commonly prescribed [6]. Although corticosteroids are effective in controlling manifestations of cGVHD [7], treatment-emergent adverse effects (TEAEs), including immunosuppression (increased susceptibility to infection) and osteoporosis, may occur [8–10]. In addition, up to approximately 50% of patients develop steroid-dependent or steroid-refractory cGVHD (or both) and require additional treatment [4, 11–14].

Ibrutinib is a first-in-class oral Bruton’s tyrosine kinase (BTK) inhibitor that is administered once daily [15]. Its mechanism of action is blockade of downstream immune receptor activation [16], thereby irreversibly inhibiting BTK. An accompanying benefit is that ibrutinib also irreversibly inhibits interleukin-2-inducible T-cell kinase (ITK) [17]. It was the first agent approved in the United States of America (USA) for the second-line treatment of cGVHD, based on results of a phase 1b/2 study in patients who had previously received 1–3 treatments for cGVHD [15]. In Japan, ibrutinib was approved, based on results of the GVH3001 study of Japanese patients, for steroid-dependent or -refractory cGVHD [11, 13]. In this study, ibrutinib had an acceptable safety profile and the best overall response rate (BORR) was 73.7% after a median of 9.6 months’ treatment (primary analysis) [11] and 84.2% after a median of 16.3 months (final analysis) [13]. However, when the results were analysed by involved organ, the response rate for those with lung involvement was lower than the overall response rate (primary analysis: 14.3% vs 73.7%; final analysis: 14.3% vs 84.2%).

Pulmonary lesions in cGVHD are a manifestation of bronchiolitis obliterans syndrome (BOS), an obstructive lung disease characterised by a decrease in forced expiratory volume in 1 s (FEV_1_) [18]. Similar to the pathophysiology of chronic rejection after lung transplantation [19], BOS is a refractory and life-threatening complication, often requiring lung transplantation in severe cases [20]. However, the 2014 National Institutes of Health (NIH) Consensus Development Project criteria that was used to evaluate response in the GVH3001 study do not include measurement of change in corticosteroid dose, or specify scales to assess lung function or symptom burden [11, 21]. As such, we hypothesised that the benefit of ibrutinib in patients with lung involvement at baseline may not have been fully captured in the pre-planned analyses of GVH3001. The present *post hoc* analysis was undertaken because: (1) the frequency of lung involvement at the onset of cGVHD after transplant is high [5]; (2) moderate-to-severe lung lesions can be difficult to manage in this patient population [5]; (3) the lung response rate is markedly lower than the overall response rate [11]; and (4) well-known TEAEs can occur following prolonged corticosteroid treatment [10]. The objectives of this descriptive analysis were to better understand the temporal change of the daily corticosteroid dose and to describe the change in lung function and symptom burden during ibrutinib treatment, specifically among patients with lung involvement at baseline in the GVH3001 study.

## Materials and methods

### Study design and patients

This was a *post hoc* subgroup analysis of data from the GVH3001 study (ClinicalTrials.gov identifier: NCT03474679) [11, 13]. The design of the GVH3001 study has been described in detail in the previously-reported primary and final analyses [11, 13]. The protocol of the GVH3001 study was approved by the Investigational Review Board of each involved study site, and the study was conducted in accordance with the ethical principles of the Declaration of Helsinki consistent with the International Council for Harmonization guidelines on Good Clinical Practices and local regulatory guidelines. Informed consent was obtained from all patients prior to their enrolment in the GVH3001 study.

### *Post hoc* analyses

All patients in the all-treated population were included in this *post hoc* analysis. Patient demographic and clinical characteristics, treatment history, ibrutinib exposure, median time in the study and treatment response were compared between patients with and without lung involvement at baseline. Treatment response was evaluated by calculating the BORR, defined as patients who had a complete response (CR) or partial response (PR) according to the NIH Consensus Development Project on Criteria for Clinical Trials in the cGVHD report [22].

In the subgroup with lung involvement, we evaluated the changes in daily corticosteroid dose (using prednisolone-equivalent doses), and effectiveness endpoints (lung function and symptom burden). Lung function was assessed based on the percent predicted FEV_1_ (%FEV_1_). The lung-related symptom burden was evaluated using the lung subscale score from the Lee cGVHD Symptom Scale [23] in the subgroup of patients with lung involvement at baseline. On this subscale, a higher score reflects worsening symptoms [23].

### Statistical analysis

Descriptive statistics were used to summarise the data, including the number of patients, arithmetic mean, standard deviation, median, minimum and maximum for continuous variables and the number and percentage of patients for categorical variables. For the BORR, 95% confidence intervals (CIs) were calculated using the Clopper-Pearson method. All statistical analyses were conducted using SAS Version 9.4 (SAS Institute Inc., Cary, North Carolina, USA).

## Results

### Patient population

All 19 patients who received ibrutinib in the GVH3001 study were included in the present analysis; seven patients had lung involvement at baseline (Table 1). In the overall patient population, the median age was 40.0 years (the study included one adolescent patient aged 13 years) and 63.2% of patients were men.

**Table 1:** Patient characteristics

	Overall (N = 19)	Lung involvement (n = 7)	No lung involvement (n = 12)
Age, years, median (range)	40.0 (13–64)	40.0 (13–53)	41.0 (20–64)
Age group, years, n (%)			
≤ 17	1 (5.3)	1 (14.3)	0
≥ 18 to ≤ 64	18 (94.7)	6 (85.7)	12 (100)
Sex, n (%)			
Female	7 (36.8)	3 (42.9)	4 (33.3)
Male	12 (63.2)	4 (57.1)	8 (66.7)
Haemoglobin, g/L, median (range)	130.0 (84–161)	129.0 (84–161)	132.0 (89–152)
Platelet count, × 10^9^/L, median (range)	162.0 (44–294)	162.0 (61–246)	170.0 (44–294)
Absolute neutrophil count, × 10^9^/L, median (range)	4.9 (0.9–8.6)	6.5 (3.0–8.6)	4.4 (0.9–7.7)
Bilirubin, µmol/L, median (range)	6.8 (4.8–17.1)	6.8 (4.8–11.3)	7.7 (5.1–17.1)
cGVHD disease state, n (%)			
Steroid dependent	10 (52.6)	4 (57.1)	6 (50.0)
Steroid refractory	9 (47.4)	3 (42.9)	6 (50.0)
Overall severity of cGVHD, n (%)			
Moderate	10 (52.6)	3 (42.9)	7 (58.3)
Severe	9 (47.4)	4 (57.1)	5 (41.7)
Time from last transplantation to enrolment, months, median (range)	36.9 (9.3–162.6)	33.4 (19.0–145.4)	39.8 (9.3–162.6)
Time from initial cGVHD diagnosis date, months, median (range)	21.4 (5.1–158.0)	18.4 (5.1–141.8)	29.9 (6.0–158.0)
Time from transplantation to initial cGVHD diagnosis date, months, median (range)	8.3 (3.4–22.8)	9.7 (3.6–22.8)	7.9 (3.4–22.6)
Karnofsky/Lansky performance status score, n (%)			
100	2 (10.5)	0	2 (16.7)
90	5 (26.3)	0	5 (41.7)
70–80	12 (63.2)	7 (100)	5 (41.7)
Number of prior cGVHD treatment regimens, median (range)	2.0 (1–3)	2.0 (1–3)	1.5 (1–3)
Prior cGVHD treatment regimens, n (%)			
1	8 (42.1)	2 (28.6)	6 (50.0)
2	9 (47.4)	4 (57.1)	5 (41.7)
3	2 (10.5)	1 (14.3)	1 (8.3)
Number of organs involved in cGVHD, n (%)			
1	1 (5.3)	0	1 (8.3)
2	7 (36.8)	2 (28.6)	5 (41.7)
3	1 (5.3)	0	1 (8.3)
4	4 (21.1)	1 (14.3)	3 (25.0)
5	3 (15.8)	1 (14.3)	2 (16.7)
6	1 (5.3)	1 (14.3)	0
7	2 (10.5)	2 (28.6)	0
Organs involved in cGVHD, n (%)			
Skin	14 (73.7)	6 (85.7)	8 (66.7)
Nails	2 (10.5)	1 (14.3)	1 (8.3)
Scalp and body hair	1 (5.3)	1 (14.3)	0
Mouth	15 (78.9)	5 (71.4)	10 (83.3)
Eyes	9 (47.4)	4 (57.1)	5 (41.7)
Genitalia	2 (10.5)	0	2 (16.7)
GI tract	6 (31.6)	2 (28.6)	4 (33.3)
Liver	2 (10.5)	2 (28.6)	0
Lung	7 (36.8)	7 (100)	0
Muscles/fascia/joints	10 (52.6)	4 (57.1)	6 (50.0)
Other	1 (5.3)	1 (14.3)	0
Corticosteroid dose (prednisolone equivalent), mg/kg/day, median (range)	0.3 (0.1–1.8)	0.4 (0.1–1.8)	0.3 (0.1–0.7)

*cGVHD* chronic graft-versus-host disease; *GI* gastrointestinal

There were several differences in demographic and baseline characteristics between patients who had lung involvement (n = 7) and those who did not (n = 12; Table 1). Patients who had lung involvement had higher median baseline neutrophil counts (6.5 × 10^9^/L) compared with patients who did not have lung involvement (4.4 × 10^9^/L). In addition, all patients who had lung involvement had a Karnofsky/Lansky performance status score of 70–80 (lower scores reflect greater functional impairment), while among patients who did not have lung involvement, 41.7% had performance status scores of 70–80 and 58.3% had scores of 90 and above. Furthermore, patients with lung involvement tended to have a greater number of organs affected than patients without lung involvement at baseline. Lastly, the median dose of corticosteroids at baseline was higher in patients with lung involvement than in those without lung involvement (0.4 vs 0.3 mg/kg/day, respectively).

All patients had received treatment for cGVHD before entering the GVH3001 study (Supplemental Table S1). In addition to corticosteroids (prednisolone), which was administered in 100% of patients with and without lung involvement, the most common treatments were tacrolimus hydrate (in 85.7% vs 91.7%, respectively) and mycophenolate mofetil (28.6% vs 25.0%, respectively).

### Ibrutinib exposure and response

In the overall patient population, the median duration of ibrutinib treatment in the GVH3001 study was 16.3 months (Supplemental Table S2). The median treatment duration was shorter in patients with versus without lung involvement (9.6 vs 17.3 months, respectively). The median absolute dose intensity of ibrutinib was lower in patients with versus without lung involvement (388.2 vs 412.2 mg/day). The median relative dose intensity was 93.3% in patients with lung involvement and 99.0% in patients without lung involvement. In the overall patient population, the median time in the study was 31.1 months. The median time in the study was shorter in patients with lung involvement than in patients without lung involvement (10.8 vs 33.7 months, respectively).

In the overall patient population, 16 patients had CR or PR, resulting in a BORR of 84.2% (95% CI 60.4–96.6%; Supplemental Table S3). With regard to the best overall whole-body assessment of the patients with lung involvement, six had PR and one had stable disease (SD), resulting in a BORR of 85.7% (95% CI 42.1–99.6%; Supplemental Table S3). Among patients without lung involvement, 10 had CR or PR, resulting in a BORR of 83.3% (95% CI 51.6–97.9%; Supplemental Table S3). In patients with lung involvement, the best overall response reported in the lungs was CR in one patient and SD in six patients (Table 2).

**Table 2:** Best overall response by organ in patients with lung involvement

Patient	Skin	Eye	Mouth	Oesophagus	Upper GI	Lower GI	Liver	Lung	Joints and fascia	Overall response
1	SD	PR	SD	NA	NA	NA	NA	SD	SD	PR
2	SD	NA	NE	NA	NA	NA	NA	SD	NA	SD
3	SD	SD	SD	SD	NA	NA	NA	SD	PR	PR
4	NA	NA	NA	NE	NA	NA	PR	SD	NA	PR
5	SD	NA	CR	CR	CR	CR	PR	SD	PR	PR
6	PR	SD	SD	NA	NA	NA	NA	CR	PR	PR
7	CR	PR	CR	NA	NA	NA	NA	SD	NA	PR

*CR* complete response; *GI* gastrointestinal; *NA* not available; *NE* not evaluable; *PR* partial response; *SD* stable disease

### Changes in the dose of corticosteroids in patients with lung involvement

**Fig. 1:**
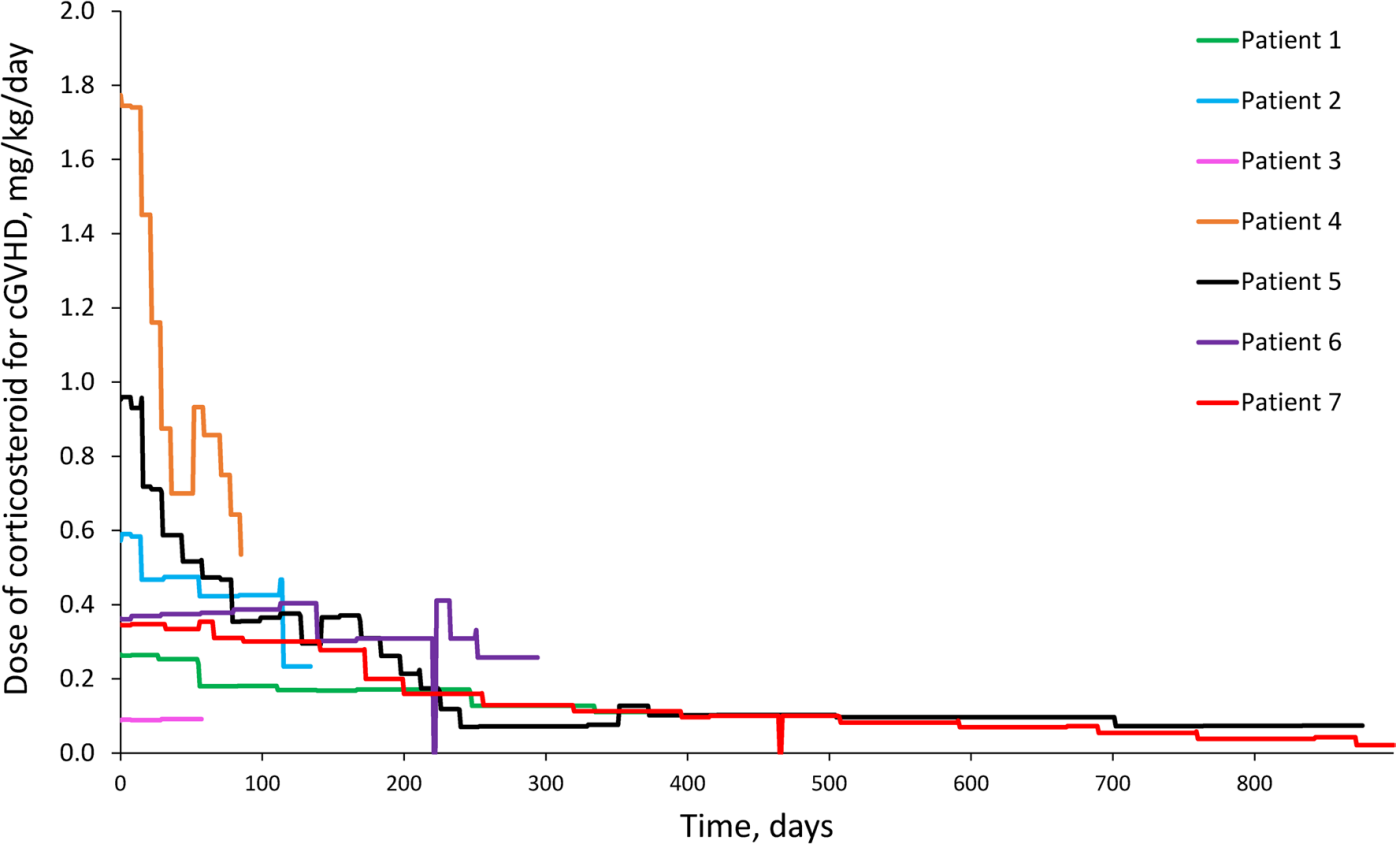
Prednisolone-equivalent corticosteroid dose used to treat cGVHD only, in patients with lung involvement in the GVH3001 study. Day 0 indicates baseline. *cGVHD* chronic graft-versus-host disease

The corticosteroid administration period in the subgroup of patients with lung involvement in the GVH3001 study ranged from 57 to 1102 days when corticosteroids were used for any indication (Supplemental Fig. 1) and from 57 to 898 days when corticosteroids were used for cGVHD only (Fig. 1). The prednisolone-equivalent corticosteroid dose used to manage cGVHD only, tended to decrease over time in almost all patients with lung involvement, the exceptions being Patient 3, for whom data were collected for the shortest period in GVH3001, and Patient 6, whose prednisolone-equivalent corticosteroid dose did not change, except for a temporary halt of corticosteroid administration shortly after day 200 (Fig. 1). This occurred due to a temporary loss of appetite, which made oral ingestion of drugs difficult at the time; Patient 6 had thus skipped corticosteroid doses for 2 days, and after recovery of the temporary illness, corticosteroid doses were resumed. In addition, Patient 6 was the only patient to achieve CR in the lungs and they had received corticosteroids for 294 days. Patient 7, who was monitored for the longest period (898 and 1102 days, regarding corticosteroids use for cGVHD only and for any indication, respectively), had received corticosteroids both for cGVHD and to temporarily manage a grade 3 anaphylactic reaction that developed on Day 464. The suspected cause of anaphylaxis was meropenem used to treat bronchial pneumonia, and the prednisolone-equivalent dose administered for this event was the highest corticosteroid dose recorded in this subgroup (Supplemental Fig. 1). The prednisolone-equivalent corticosteroid dose fluctuated over the course of the study, regardless of its intended use in each patient with lung involvement (Supplemental Fig. 1).

### Changes in lung function and symptom burden in patients with lung involvement

Changes in %FEV_1_ over the course of the study remained relatively stable in Patients 5 and 7, and increased to within the normal range (> 80%) in Patient 6 (Supplemental Fig. S2). Due to the short duration of follow-up, no conclusions could be drawn about the changes in %FEV_1_ in Patients 2, 3 and 4. Changes in the lung subscale score on the Lee cGVHD Symptom Scale remained relatively stable throughout the study (Supplemental Fig. S3).

### TEAEs

The most frequently-experienced TEAEs (> 25%) in the safety analysis population were pneumonia and stomatitis, (47.4% each), cellulitis, platelet count decreased, and upper respiratory tract infection (31.6% each) and nausea (26.3%; Table 3). Interstitial-lung disease (ILD; reported as bronchiolitis in this study) was reported in two patients (10.5%), both of whom had lung involvement at baseline. Both cases were due to infections and were considered unrelated to ibrutinib treatment. There were no notable differences in the occurrence of TEAEs between patients with and without lung involvement at baseline.

**Table 3:** Occurrence of one or more treatment-emergent adverse event (TEAE) in the safety analysis set

Safety analysis set	Overall		Lung involvement		No lung involvement	
	(N = 19)		(n = 7)		(n = 12)	
n (%)	All grades	Grade ≥ 3	All grades	Grade ≥ 3	All grades	Grade ≥ 3
Patients with ≥ 1 TEAE	19 (100.0)	17 (89.5)	7 (100.0)	7 (100.0)	12 (100.0)	10 (83.3)
Preferred term						
Pneumonia	9 (47.4)	6 (31.6)	4 (57.1)	3 (42.9)	5 (41.7)	3 (25)
Stomatitis	9 (47.4)	2 (10.5)	4 (57.1)	0	5 (41.7)	2 (16.7)
Cellulitis	6 (31.6)	3 (15.8)	2 (28.6)	1 (14.3)	4 (33.3)	2 (16.7)
Platelet count decreased	6 (31.6)	3 (15.8)	3 (42.9)	1 (14.3)	3 (25)	2 (16.7)
Upper respiratory tract infection	6 (31.6)	0	3 (42.9)	0	3 (25)	0
Nausea	5 (26.3)	0	1 (14.3)	0	4 (33.3)	0
Cataract	4 (21.1)	2 (10.5)	1 (14.3)	1 (14.3)	3 (25)	1 (8.3)
Constipation	4 (21.1)	0	1 (14.3)	0	3 (25)	0
Headache	4 (21.1)	0	0	0	4 (33.3)	0
Oedema peripheral	4 (21.1)	0	0	0	4 (33.3)	0
Pruritus	4 (21.1)	0	2 (28.6)	0	2 (16.7)	0
Purpura	4 (21.1)	0	1 (14.3)	0	3 (25)	0
Abdominal pain upper	3 (15.8)	0	1 (14.3)	0	2 (16.7)	0
Anaemia	3 (15.8)	2 (10.5)	1 (14.3)	0	2 (16.7)	2 (16.7)
Anxiety	3 (15.8)	0	1 (14.3)	0	2 (16.7)	0
Arthralgia	3 (15.8)	0	0	0	3 (25)	0
Conjunctivitis	3 (15.8)	0	2 (28.6)	0	1 (8.3)	0
Cough	3 (15.8)	0	1 (14.3)	0	2 (16.7)	0
Diarrhoea	3 (15.8)	0	0	0	3 (25)	0
Epistaxis	3 (15.8)	0	0	0	3 (25)	0
Hypertension	3 (15.8)	1 (5.3)	1 (14.3)	1 (14.3)	2 (16.7)	0
Muscle spasms	3 (15.8)	0	1 (14.3)	0	2 (16.7)	0
Nasopharyngitis	3 (15.8)	0	0	0	3 (25)	0
Paronychia	3 (15.8)	0	1 (14.3)	0	2 (16.7)	0
Pleural effusion	3 (15.8)	1 (5.3)	1 (14.3)	1 (14.3)	2 (16.7)	0
Bronchiolitis	2 (10.5)	1 (5.3)	2 (28.6)	1 (14.3)	0	0

## Discussion

This descriptive *post hoc* analysis of the GVH3001 study showed that the daily corticosteroid dose decreased over time in five out of seven patients with cGVHD and lung involvement (Patients 1, 2, 4, 5, and 7). Corticosteroid use could not be determined in Patient 3 due to their short duration of treatment. The best overall response to ibrutinib in the lung was CR in Patient 6, who was the only patient to achieve CR, possibly because of better baseline % FEV_1_ and Lee cGVHD Symptom Scale lung subscale scores in comparison to the other patients, suggesting that pulmonary symptoms were mild at baseline. Apart from the 2-day interruption in corticosteroid dosing in Patient 6, the administration of corticosteroids remained unchanged. Thus, we did not consider that corticosteroid use had decreased due to an improvement of cGVHD in this case. In addition, the overall effectiveness of ibrutinib was maintained in six out of seven patients (i.e., all except Patient 3). Improvements in standard assessments of clinical benefit such as treatment response rate were reported and there was no progression of lung cGVHD during the study period in these patients. At the same time, lung function (%FEV_1_) and the lung subscale score on the Lee cGVHD Symptom Scale remained relatively stable, suggesting that the reduction in the daily corticosteroid dose was not accompanied by deterioration of lung function or increases in lung symptom burden.

The findings of the present analysis are in line with published literature. Since TEAEs due to corticosteroid use are known to negatively affect QoL [2, 10], it is beneficial to reduce the daily corticosteroid dose in patients with cGVHD who are receiving ibrutinib treatment. The effect of ibrutinib on daily corticosteroid dose was evaluated using Lee cGVHD Symptom Scale scores (lung subscale) in a phase 1b/2, open-label, single-arm study conducted in 42 patients with steroid-dependent or -refractory cGVHD who had received up to three previous treatments [14]. Over the median follow-up of 26 months, the corticosteroid dose decreased to < 0.2 mg/kg/day in 64% of patients [14]. Among patients who had CR or PR, the median corticosteroid dose decreased from 0.3 mg/kg/day at baseline (data were available for 29 patients) to 0.1 mg/kg/day at week 52 (data were available for 18 patients) [14]. In contrast, a retrospective, single-centre database study found that ibrutinib was not associated with a reduction in corticosteroid dose between initiation of the drug and failure-free survival event or last follow-up in 53 patients with steroid-refractory cGVHD. However, the average prednisone-equivalent dose was low (0.26 mg/kg/day) at ibrutinib initiation, which would have made it difficult to discern any decrease in dose over the course of the study [24].

A clinically meaningful improvement in the Lee cGVHD Symptom Scale was observed in the phase 1b/2 study at week 52 in 16 out of 29 patients who had CR or PR and in one patient who did not have either CR or PR [14]. The effect of ibrutinib on lung function was evaluated in a study conducted in 17 patients with steroid-dependent or -refractory cGVHD and lung involvement who had received an average of four previous treatments [25]. At 180 days, %FEV_1_ increased in seven out of 14 patients who continued to receive ibrutinib, and lung function was stable during ibrutinib treatment in most patients [25].

As previously mentioned, the response rate to ibrutinib in the lungs was notably lower than the overall response rate in the primary and final analyses of the GVH3001 study [11, 13]. Our *post hoc* analysis showed that patients who had lung involvement tended to have more severe disease at baseline than those without lung involvement, with higher absolute neutrophil counts, worse Karnofsky/Lansky performance status, involvement across a wider variety of organs, as well as involvement of a greater number of organs, and a higher daily corticosteroid dose. It should be noted that high neutrophil counts may reflect the fact that many people on high doses of corticosteroids have pulmonary complications. It is also possible that high doses of corticosteroids release neutrophils attached to peripheral blood vessels, resulting in high neutrophil counts; however, this mechanism and any possible association between raised neutrophil counts and the effects of ibrutinib are yet to be fully elucidated. Nevertheless, despite having more severe disease at baseline, there was no notable difference in the overall BORR of ibrutinib between patients with lung involvement and those without (85.7% vs 83.3%, respectively), suggesting that the low pulmonary efficacy observed in the primary analysis was not predictive of the overall efficacy of ibrutinib.

There were no notable differences in the occurrence of TEAEs between patients with and without lung involvement, except for the occurrence of ILD (reported as bronchiolitis in this study) in two patients (both with lung involvement at baseline). Neither case was judged to have been drug-induced ILD as they were both due to infections. Ibrutinib has been reported to cause ILD in a small proportion of patients [11], however, the mechanism has not been elucidated. It remains important to balance the effectiveness of ibrutinib treatment with the associated risks when selecting treatment for patients with cGVHD and lung involvement.

The present analysis had several limitations. Specifically, the number of patients with lung involvement in the GVH3001 study was small, and for some patients, corticosteroid dosage data were limited to a relatively short duration, making it difficult to draw conclusions about the effect of ibrutinib on the dose of corticosteroids.

In conclusion, this *post hoc* analysis of the GVH3001 study showed that, in some previously treated patients with cGVHD and associated lung involvement, ibrutinib may allow for corticosteroid dose reduction without worsening of lung function or an increase in lung symptom burden. As prolonged corticosteroid treatment is associated with significant TEAEs, this represents a notable clinical benefit of ibrutinib in patients with cGVHD and pulmonary symptoms. While data from this study are encouraging, further research is needed in larger cohorts to elucidate the role of ibrutinib in this specific patient population.

## Supplementary Information

Below is the link to the electronic supplementary material.

Supplementary file1 (DOCX 191 KB)

## Data Availability

The data sharing policy of Janssen Pharmaceutical Companies of Johnson & Johnson is available at https://www.janssen.com/clinical-trials/transparency. As noted on this site, requests for access to the study data can be submitted through the Yale Open Data Access (YODA) Project site at http://yoda.yale.edu.
