# Supplementary material for: Temporal changes in corticosteroid dose during ibrutinib treatment in patients with cGVHD and pulmonary involvement
Source: Int J Hematol. 2024 Dec 10;121(3):388–96. doi: 10.1007/s12185-024-03882-1 (PMC11861523; doi:10.1007/s12185-024-03882-1)

**Temporal changes in corticosteroid dose during ibrutinib treatment in patients with cGVHD and pulmonary involvement**

**Authors:** Masako Toyosaki,^1^ Shinichiro Machida,^1^ Daisuke Tomizawa,^2^ Masaya Okada,^3^ Masashi Sawa,^4^ Yasunori Ueda,^5^ Ai Omi,^6^ Yosuke Koroki,^6^ Takanori Teshima^7^

**Affiliations:** ^1^Department of Hematology and Oncology, Tokai University Hospital, Kanagawa, Japan; ^2^Division of Leukemia and Lymphoma, Children's Cancer Center, National Center for Child Health and Development, Tokyo, Japan; ^3^Division of Hematology and Oncology, Kansai Medical University General Center, Osaka, Japan; ^4^Department of Hematology and Oncology, Anjo Kosei Hospital, Anjo, Japan; ^5^Department of Hematology/Oncology, Kurashiki Central Hospital, Kurashiki, Japan; ^6^Medical Affairs Division, Janssen Pharmaceutical K.K., Tokyo, Japan; ^7^Department of Hematology, Faculty of Medicine, Hokkaido University, Sapporo, Japan.

**Corresponding author:** Ai Omi, Medical Affairs Division, Janssen Pharmaceutical K.K, 3-5-2 Nishi-kanda, Chiyoda-ku, Tokyo 101-0065, Japan. E-mail: AOmi@ITS.JNJ.com

# Supplementary materials

## SUPPLEMENTAL TABLE S1.

Previous treatments for cGVHD.

| **n (%)** | **Overall (N = 19)** | **Lung involvement (n = 7)** | **No lung involvement (n = 12)** |
| --- | --- | --- | --- |
| Any previous cGVHD treatment | 19 (100) | 7 (100) | 12 (100) |
| Prednisolone | 19 (100) | 7 (100) | 12 (100) |
| Tacrolimus hydrate | 17 (89.5) | 6 (85.7) | 11 (91.7) |
| Mycophenolate mofetil | 5 (26.3) | 2 (28.6) | 3 (25.0) |
| Methotrexate | 2 (10.5) | 2 (28.6) | 0 |
| Cyclosporin | 1 (5.3) | 1 (14.3) | 0 |
| Extracorporeal photopheresis | 1 (5.3) | 0 | 1 (8.3) |
| Rituximab | 1 (5.3) | 0 | 1 (8.3) |
| Teceleukin | 1 (5.3) | 0 | 1 (8.3) |

cGVHD, chronic graft-versus-host disease.

## SUPPLEMENTAL TABLE S2.

Exposure to ibrutinib during the GVH3001 study.

|  | **Overall (N = 19)** | **Lung involvement (n = 7)** | **No lung involvement (n = 12)** |
| --- | --- | --- | --- |
| Treatment duration, months, median (range)^a^ | 16.3 (0.6–36.2) | 9.6 (1.9–36.2) | 17.3 (0.6–36.2) |
| Cumulative total dose, mg, median (range) | 96880.0 (7980–457380) | 68040.0 (23940–367920) | 204120.0 (7980–457380) |
| Dose intensity, mg/day, median (range)^b^ | 409.9 (157.8–420.0) | 388.2 (232.2–420.0) | 412.2 (157.8–420.0) |
| Relative dose intensity, %, mean ± SD^c^ | 89.3 ± 14.2 | 91.6 ± 9.3 | 87.9 ± 16.6 |
| Relative dose intensity, %, median (range)^c^ | 98.3 (50.1–100.0) | 93.3 (75.6–100.0) | 99.0 (50.1–100.0) |
| Time in the study, months, median (range)^d^ | 31.1 (1.9–38.6) | 10.8 (1.9–38.5) | 33.7 (17.9–38.6) |

^a^Treatment duration was defined as (date of last dose of study drug – date of first dose of study drug + 1 day) / 30.4375.

^b^Dose intensity was calculated as (sum of total daily dose during the treatment phase) / study drug duration.

^c^Relative dose intensity was calculated by total cumulative dose administered / total expected dose × 100%.

^d^Time in the study was defined as (study exit date/last known alive date – date of first dose of study drug + 1 day) / 30.4375.

SD, standard deviation.

## SUPPLEMENTAL TABLE S3.

Best overall response rate by organ.

|  | **Overall (N = 19)** | | | **Lung involvement (n = 7)** | | | **No lung involvement (n = 12)** | | |
| --- | --- | --- | --- | --- | --- | --- | --- | --- | --- |
|  | **n** | **Organ response rate; PR + CR, n (%)^a^** | **95% CI^b^** | **n** | **Organ response rate; PR + CR, n (%)^a^** | **95% CI^b^** | **n** | **Organ response rate; PR + CR, n (%)^a^** | **95% CI^b^** |
| Skin | 14 | 8 (57.1) | 28.9–82.3 | 6 | 2 (33.3) | 4.3–77.7 | 8 | 6 (75.0) | 34.9–96.8 |
| Eye | 9 | 2 (22.2) | 2.8–60.0 | 4 | 2 (50.0) | 6.8–93.2 | 5 | 0 | 0.0–52.2 |
| Mouth | 15 | 6 (40.0) | 16.3–67.7 | 5 | 2 (40.0) | 5.3–85.3 | 10 | 4 (40.0) | 12.2–73.8 |
| Oesophagus | 5 | 3 (60.0) | 14.7–94.7 | 2 | 1 (50.0) | 1.3–98.7 | 3 | 2 (66.7) | 9.4–99.2 |
| Upper GI | 3 | 2 (66.7) | 9.4–99.2 | 1 | 1 (100.0) | 2.5–100.0 | 2 | 1 (50.0) | 1.3–98.7 |
| Lower GI | 2 | 2 (100.0) | 15.8–100.0 | 1 | 1 (100.0) | 2.5–100.0 | 1 | 1 (100.0) | 2.5–100.0 |
| Liver | 2 | 2 (100.0) | 15.8–100.0 | 2 | 2 (100.0) | 15.8–100.0 | 0 | - | - |
| Lung | 7 | 1 (14.3) | 0.4–57.9 | 7 | 1 (14.3) | 0.4–57.9 | 0 | - | - |
| Joints and fascia | 10 | 6 (60.0) | 26.2–87.8 | 4 | 3 (75.0) | 19.4–99.4 | 6 | 3 (50.0) | 11.8–88.2 |
| Overall response | 19 | 16 (84.2) | 60.4–96.6 | 7 | 6 (85.7) | 42.1–99.6 | 12 | 10 (83.3) | 51.6–97.9 |

^a^Response rate was calculated as number of patients with partial + complete response / number of patients with organ involvement × 100%.

^b^Two-sided 95% CI was calculated using the Clopper-Pearson exact method.

CI, confidence interval; CR, complete response; GI, gastrointestinal, PR, partial response.

## SUPPLEMENTAL FIGURE S1.

Prednisolone-equivalent corticosteroid dose used to treat chronic graft versus host disease (cGVHD) and adverse events in patients with lung involvement during the GVH3001 study. Day 0 indicates baseline.


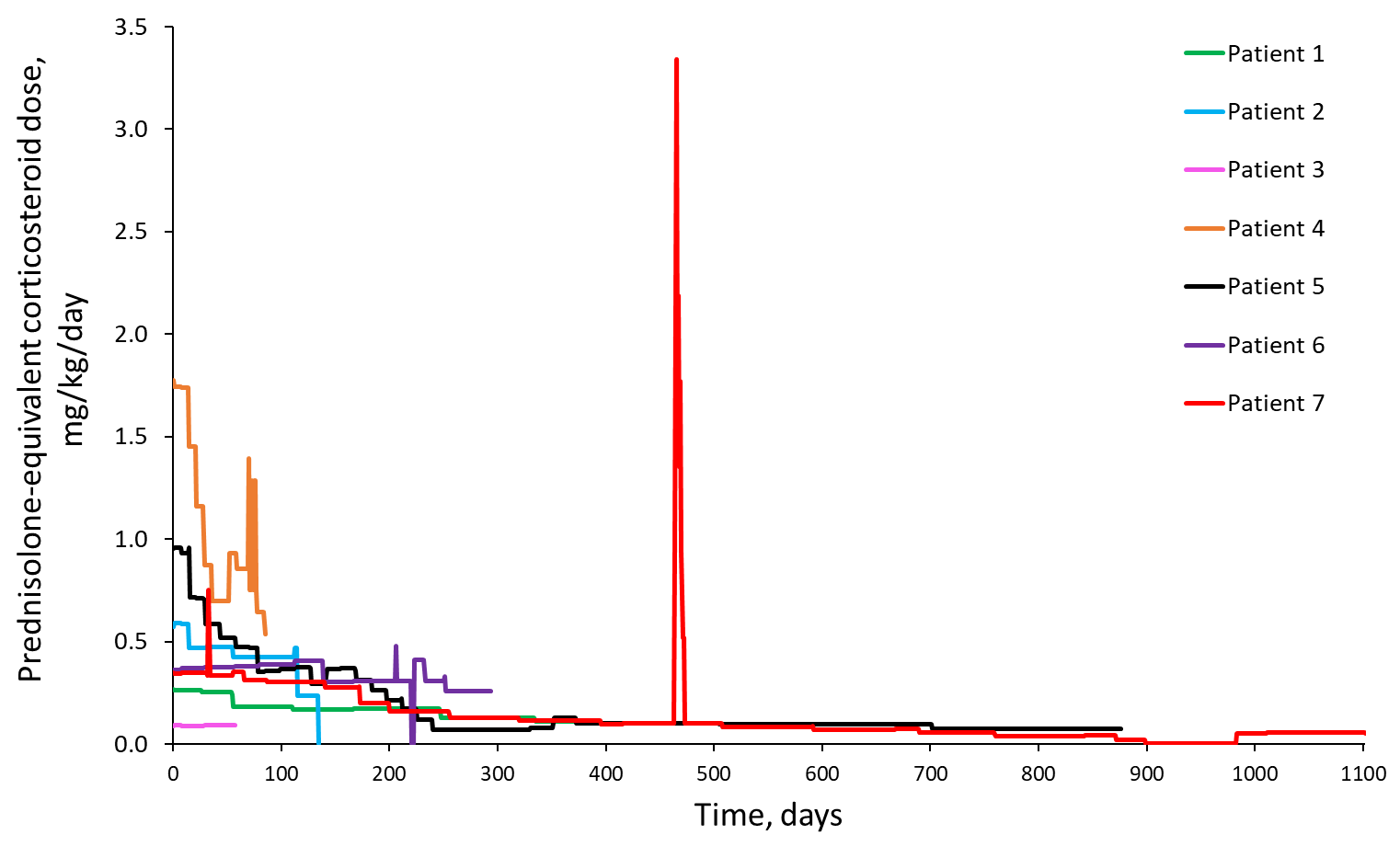


## SUPPLEMENTAL FIGURE S2.

%FEV1 over time in patients with lung involvement during the GVH3001 study. Day 0 indicates baseline. %FEV1, percent predicted forced expiratory volume in 1 second.


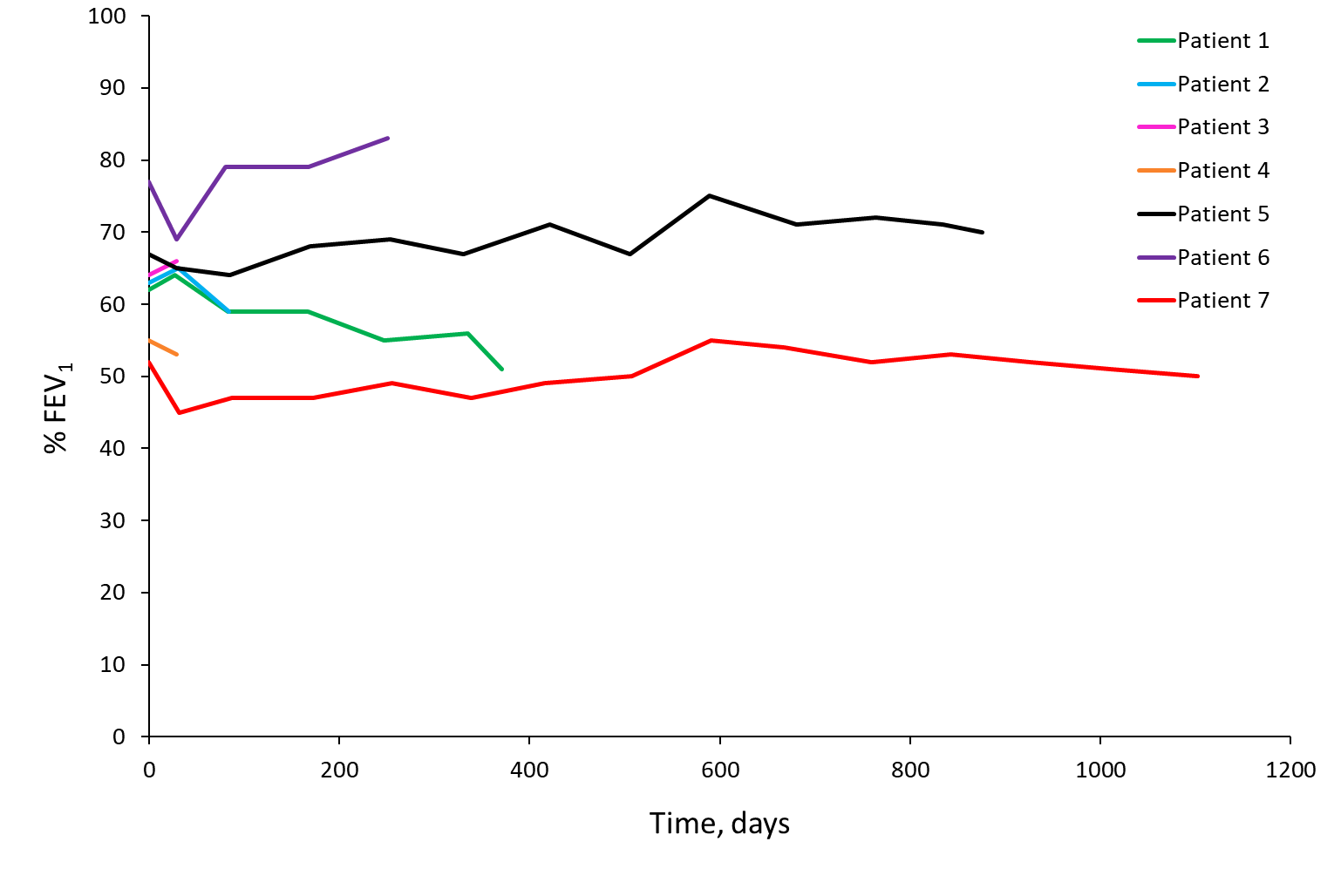


## SUPPLEMENTAL FIGURE S3.

Lee cGVHD Symptom Scale lung subscale scores over time during the GVH3001 study in patients with lung involvement. Higher scores reflect worsening symptoms. Day 0 indicates baseline. cGVHD, chronic graft-versus-host disease.


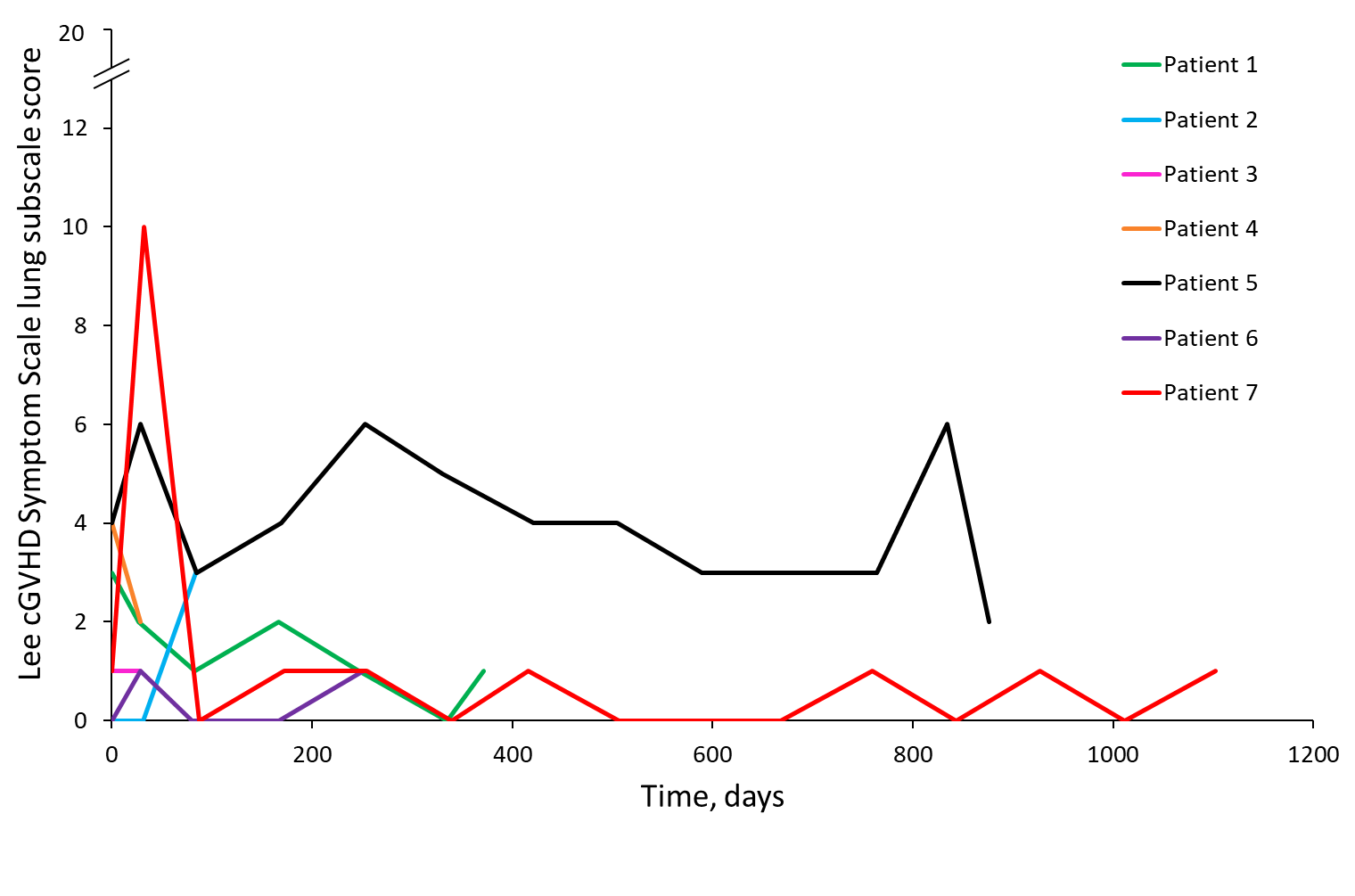

Supplement: Supplementary file 1 — Supplementary file1 (DOCX 191 KB) [file 12185_2024_3882_MOESM1_ESM.docx]
